# Individuals with peripheral vestibulopathy and poor quality of sleep are at a higher risk for falls

**DOI:** 10.1016/j.bjorl.2019.10.013

**Published:** 2019-12-10

**Authors:** Mario Chueire de Andrade Junior, Renato Stefanini, Juliana Maria Gazzola, Fernanda Louise Martinho Haddad, Fernando Freitas Ganança

**Affiliations:** Universidade Federal de São Paulo, Escola Paulista de Medicina, Departamento de Otorrinolaringologia e Cirurgia de Cabeça e Pescoço, São Paulo, SP, Brazil

**Keywords:** Postural balance, Sleep disorders, Quality of life, Vestibular disorders

## Abstract

**Introduction:**

There is a lack of scientific studies on the assessment of patients with vestibular disorders associated with sleep quality disorders and its impact on the balance and overall quality of life.

**Objectives:**

to assess the impact of the sleep quality on the balance and quality of life of individuals with peripheral vestibulopathies.

**Methods:**

52 individuals with peripheral vestibulopathies underwent sleep quality assessment through the Pittsburgh sleep quality index, neurotological examination through dizziness handicap inventory and Tetrax posturography (Sunlight Medical Ltd.) in eight sensory conditions. Thirty-two healthy individuals (G3) participated as the control group.

**Results:**

Fourteen individuals with vestibulopathy had good quality of sleep (G1) and 38 showed poor quality of sleep (G2) as demonstrated by the Pittsburgh sleep quality index global scores (*p* = 0.001). The dizziness handicap inventory showed worse impact of the dizziness on the quality of life in G2 when compared to G1 (*p* = 0.045). The G_2_ showed higher risk of falling in posturography when compared to G3 (*p* = 0.012) and higher index of postural instability in five sensory conditions in comparison with G3. In the vestibulopathy groups, the worse the sleep quality, the higher the risk of falling (r = 0.352) and the worse the quality of life (r = 0.327).

**Conclusion:**

Individuals with peripheral vestibulopathies and poor quality of sleep demonstrate worse balance evidenced by increased postural instability, higher risk of falls and worse perceived quality of life. The quality of sleep is a predictive factor for worse perceived quality of life and for higher risk of falls in individuals with peripheral vestibulopathies.

## Introduction

Balance is the individual’s ability to stand upright and make movements with acceleration and rotation without disequilibrium or falling; it is the result of the interaction between the vestibular system, visual and proprioceptive input.^1^ One of the main tasks of the human system of postural control is to maintain balance, which is performed a on small basis of support on both feet.^2^ Concurrently with the sensory and motor systems, the vestibular system acts as a gravity sensor, helping with postural control.^2^

Maintaining balance in the upright position is a complex and continuous task in the daily life and the effects of sleep deprivation on the vestibular responses have been objects of research.^3^, ^4^, ^5^, ^6^, ^7^, ^8^ It is known that neurons taking part in the sleep stages are located in the pontine reticular formation and raphe nuclei, regions which also receive information for the otolithic organs.^9^ Dysfunctions in these organs could lead to interruptions in the sleep-wake cycle.^10^ Thus it is possible that signals coming from the vestibular system are related with sleep regulation as noted in actions such as walking, ride a car and other movements that stimulate the vestibular system and help induce sleep.^4^

Changes in the autonomic functions that include pupillary responses, heart rate and blood pressure peaks are characteristics of Rapid Eye Movement (REM) sleep and oscillations in these functions are abolished in cases of vestibular nuclei lesions.^11^ Therefore, physiological evidences indicate that the vestibular system may influence the REM sleep^12^ and that vestibular stimuli have influence on the pontine reticular formation neurons involved in the changes and the mediation between the sleep stages.^13^

Some studies focusing on the effects of sleep deprivation on the vestibular function and analyzing postural control after restricted sleep indicate that postural stability is not affected after 24 h of sleep deprivation.^14^, ^15^ While postural sway remains unchanged with sleep deprivation, it does increase when a processing of secondary information task is associated.^15^ As far as the vestibular-ocular response is concerned, only two studies have described the effects of lack of sleep on the Vestibular-Ocular Reflex (VOR). It is supposed that sleep deprivation may reduce the VOR gain but that this effect only becomes significant in cases of longer sleep deprivation periods.^5^ This reduction in the VOR gain for impulsive stimuli may be masked by the activation of a system to redirect attention after short periods of sleep deprivation.^5^

Posturography was developed to help in the analysis of the functional aspect of the underlying balance disorder, differentiating central nervous system and peripheral abnormalities of the postural control. This modality of assessment allows for the investigation of the postural control considering the difference of pressure on the platform, comparing the values originated from the anterior and posterior parts of each foot.^4^

The parameters offered by the equipment can be useful in the clinical investigation of patients with balance disorders and falls not otherwise diagnosed by the conventional test battery.^16^ Therefore, it can predict higher risk of falls, especially among the elderly, which is currently considered a public health concern and the sixth most frequent cause of death in this age group when considering its frequency and consequences.^17^

There are no known scientific studies on the assessment of patients with vestibular disorders associated with sleep quality disorders and its impact on the balance and overall quality of life.

The goal of this study is to evaluate the impact of the quality of sleep on the balance of individuals with peripheral vestibulopathies.

## Methods

The present transversal study was done in the period between January and December 2012 in the Equilibrium Assessment and Vestibular Rehabilitation Clinic of the Otology and Neurotology Discipline in the Otorhinolaryngology and Head and Neck Surgery Department of the institution, with the approval of the Ethics Committee (number 0590/11). All the participants signed the Free and Informed Consent Form.

### Participants

The sample included 52 individuals of both genders, between 18 and 80 years old, with clinical diagnosis of peripheral vestibulopathies, from the institution’s Neurotology Outpatient Clinic. The exclusion criteria was: patients presenting with any external or middle ear disease, neurological condition, difficulties in comprehension and in following simple verbal commands, inability to stand upright unassisted, serious visual impairment or visual impairment not compensated by corrective lenses, orthopedic problems that resulted in limited movement or need for prosthesis in the lower limbs, psychiatric disorders, using drugs that act on the vestibular system, that have undergone vestibular rehabilitation therapy in the last six months or who practiced any physical activity at least three times per week. In addition, 32 healthy individuals of both genders, between 18–80 years old, community dwellers, without complaints of dizziness and ear disease were included as the control group.

### Method

The assessment protocol commenced with the Pittsburgh Sleep Quality Index (PSQI) questionnaire, which evaluates the quality of sleep in a one month period. The questionnaire comprises 19 questions that generate 7 components each one scored from 0 to 3 points. When summed up, the components form a global score that varies from 0 to 21 points in which the higher the score, the worse the quality of sleep. A global score higher than 5 indicates poor quality of sleep.^18^ The Dizziness Handicap Inventory (DHI) which evaluates the interference of dizziness in the perception of the quality of life in individuals with vestibulopathies was also used in this study; the DHI is composed of 25 questions grouped into physical, functional and emotional aspects.^19^

Posturography was performed on the Tetrax Interactive Balance System (Tetrax®) by Sunlight Medical Ltd. in a quiet climatized room, dedicated to balance assessments. The equipment consists of a platform with safety handle bars and a foam mat, connected to a computer running specific software.

The individuals were instructed to stand as still and stable as possible on the platform during the 32 s time frame determined by the equipment in each one of the eight sensory conditions. The Tetrax® system provides standardized values for the Fall Index which is expressed in percentages and calculated based on the testing parameters of stability, analysis of postural sway with Fourier transformations and synchronization index for all the sensory conditions tested. The Fall Index may vary from 0 to 100 where higher scores indicate a higher risk of falling: values between 0 and 36 are considered low risk of falling; from 37 to 58, moderate risk of falling and between 59 and 100, high risk of falling.^16^

After completing the questionnaires the individuals underwent posturography, Following medical approval the individuals interrupted the use of anti-vertiginous and sleep-inducing medications for 48 prior to the assessment. The main examiner had no prior knowledge of which group each participant was part of (whether peripheral vestibular disorder or control group).

Following the analysis of the PSQI the individuals were separated into two groups classified as good quality of sleep and poor quality of sleep. The healthy individuals served as the control group to compare the posturography findings.

### Statistical analysis

The Student’s *t*-test was used to compare the averages of the two groups and when the normality of variance assumption was rejected the non-parametric Mann-Whitney test was used. For the comparison amongst the three groups the analysis of variance to a factor with multiple comparisons was used through the Bonferroni Test. When the assumption of normality was rejected the non-parametric Kruskal-Wallis one-way analysis of variance was used with the Dunn test.^20^ In order to test the homogeneity amongst proportions the qui square test or the Fisher’s exact test were applied. The Pearson’s correlation coefficient was used to study the correlation between variables.^20^ The level of significance adopted was 5 %.

## Results

The sample of this study consisted of 84 individuals of which 52 were diagnosed with peripheral vestibulopathies 44 (84.61 %) females and 8 (15.38 %) males, mean age of 60.09 ± 8.49 years, ranging from 36 to 78 years). After analyzing the PSQI scores the group with vestibulopathy was divided into G_1,_ with good quality of sleep and a global score of 5 or less, totaling 14 (27 %) patients and G_2_ with poor quality of sleep (scores higher than 5) comprised of 38 (73 %) patients. The control group (G_3_) consisted of 32 healthy volunteers. The groups were homogeneous with regards to age, weight and gender (Table 1).

**Table 1:** Average, standard deviation and absolute frequency (n) for the demographic data of the individuals with vestibulopathies and healthy individuals.

	Groups			*p*
	G_1_ (n = 14)	G_2_ (n = 38)	G_3_ (n = 32)	
Age (average, SD)	62.28 (6.59)	59.28 (9.04)	55.56 (13.44)	0.118^a^
Weight (average, SD)	72.62 (11.09)	70.51 (14.76)	66.22 (11.64)	0.229^b^
Gender				
Female, n (%)	11 (78.57)	33 (86.84)	20 (62.50)	
Male, n (%)	3 (21.43)	5 (13.16)	12 (37.50)	

a Bonferroni Analysis of Variance to a single factor.

b ANOVA.

Table 2 shows the Fall Index generated by the Tetrax® Posturography in the different groups. When the groups were compared with regards to the Fall Index a significant difference was observed. G_2_ showed significantly higher risk of falls when compared to G_3_ (*p* = 0.013); however, there were no significant differences when compared with G_1_. The groups G_1_ and G_3_ also did not show significant difference with regards to the risk of falls.

**Table 2:** Descriptive factors (average ± SD) and comparative analysis of the Fall Index (%) and the sensory conditions of the Tetrax Interactive Balance System (Tetrax®) For the 14 individuals with vestibulopathy and good quality of sleep (G_1_), 38 participants with poor quality of sleep (G_2_) and 32 healthy individuals in the control group (G_3_).

	Groups			*p*
	G_1_ (n = 14) Average (SD)	G_2_ (n = 38) Average (SD)	G_3_ (n = 32) Average (SD)	
Fall index	29.71 (25.02)	46.73 (34.07)^a^	21.31 (18.83)	0.012^a^

a Significant difference when compared with group G_3_ (Kruskall-Wallis’s test, followed by Dunn’s test, *p* < 0.05).

The assessment of the perception of the dizziness interference in the quality of life in individuals with vestibulopathies was quantified by the Dizziness Handicap Inventory (DHI). The values obtained are shown in Table 3. The group G_2_ demonstrated worse perception of the quality of life in the averages of the physical aspect (*p* = 0.018) and total score (*p* = 0.045) when compared to G_1_.

**Table 3:** Descriptive values and comparative analysis of the Dizziness Handicap Inventory (DHI) for the 14 individuals with vestibulopathy in the group with good quality of sleep (G_1_) and 38 in the group with poor quality of sleep (G_2_).

DHI	Groups	Average	Standard deviation	Minimum value	Median	Maximum value	*p*^a^
Functional	G_1_	11.00	6.74	2.00	9.00	26.00	0.326
	G_2_	13.32	7.71	0.00	12.00	32.00	
Physical	G_1_	14.29	5.54	4.00	15.00	26.00	0.018^b^
	G_2_	18.68	5.85	0.00	19.00	26.00	
Emotional	G_1_	6.86	9.40	0.00	4.00	32.00	0.076
	G_2_	11.21	6.97	0.00	10.00	28.00	
Total	G_1_	32.14	19.46	16.00	26.00	84.00	0.045^b^
	G_2_	43.21	16.30	8.00	43.00	80.00	

DHI, Dizziness Handicap Inventory.

a Student’s *t*-test.

b Significant value *p* < 0.05.

Table 4 shows the level of correlation amongst the global score on the PSQI, the Fall Index, the total DHI score and its respective functional, physical and emotional aspects for all 52 individuals with vestibulopathy. The PSQI global score showed positive and significant correlation with the Fall Index (r = 0.35), physical aspect of DHI (r = 0.34) and total DHI (r = 0.30). Thus, the higher the PSQI global score, the higher the Fall Index, DHI physical and total.

**Table 4:** Level of correlation amongst the global score on the Pittsburgh Sleep Quality Index (PSQI), the Fall Index, the total Dizziness Handicap Inventory (DHI) score and separate functional, physical and emotional scores for the 52 individuals with vestibulopathy.

	Fall index	DHI functional	DHI physical	DHI emotional	DHI total
R	0.35	0.17	0.34	0.26	0.30
*p*^a^	0.010^b^	0.221	0.013^b^	0.056	0.026^b^

DHI, Dizziness Handicap Inventory.

a Pearson’s Correlation Coefficient.

b Significant value *p* < 0.05.

**Figure 1:**
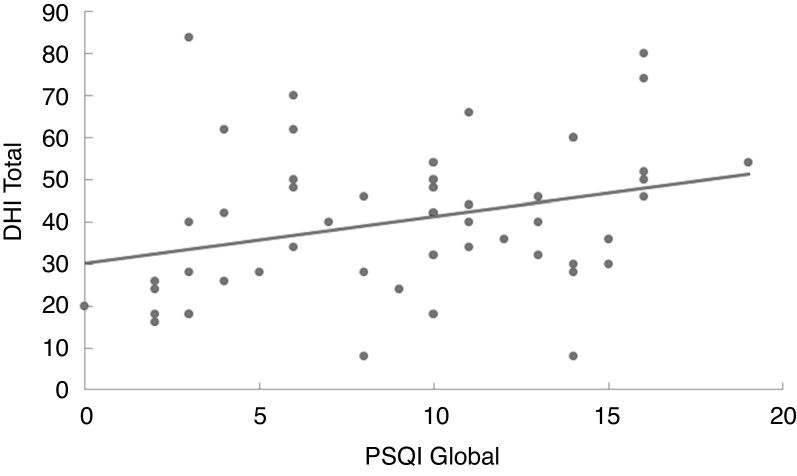
Linear regression model of the correlation between the Pittsburgh Sleep Quality Index (PSQI) global score and the Dizziness handicap Inventory (DHI).

**Figure 2:**
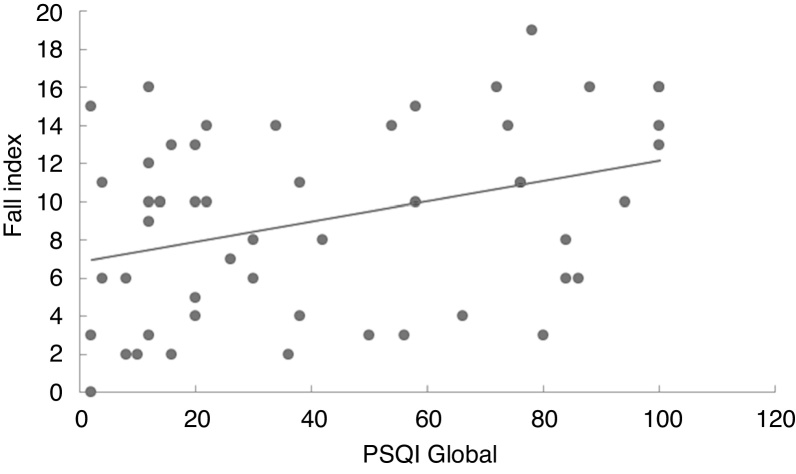
Linear regression model of the correlation between the Pittsburgh Sleep Quality Index (PSQI) global score and the Fall Index.

In the linear regression analysis of data amongst the Pittsburgh Sleep Quality Index score, the Dizziness Handicap Inventory total score (Fig. 1) and the likelihood of falls (Fig. 2) it can be noted that the quality of sleep is a predictive factor of worse quality of life (*p* = 0.027) and higher risk of falls (*p* = 0.011). Thus, the worse the quality of sleep, the worse the perception of quality of life and the higher the risk of falls in individuals with vestibulopathies.

## Discussion

The posturography assessment performed with the Tetrax Interactive Balance System (Tetrax®) showed abnormalities in the parameters of postural control analyzed in the individuals with vestibulopathy with good and poor quality of sleep. The significant results obtained in the present study demonstrate worse quality of life and higher risk of falls in the individuals with vestibulopathy when the individuals with poor quality of sleep were compared with the control group, as evidenced by a higher Fall Index and global stability score. The group with vestibulopathy and good quality of sleep seemed to perform at an intermediate level.

The prevalence of the female gender in this study corroborates other reports of higher occurrence of vestibulopathies in women, both in the community and in outpatient clinics.^21^, ^22^, ^23^, ^24^ This prevalence may be attributable to the association between vestibular diseases and hormonal imbalances and metabolic diseases typically observed in females, as much as with an increased tendency of seeking medical attention displayed by women.^25^, ^26^ Vestibulopathies may affect children and adolescents but are predominant in adults and seniors.^27^ In this study the mean age in the group with vestibulopathy was 60.09 years, ranging from 35 to 78 years, which is similar to other reports in the literature.^28^, ^29^, ^30^

The PSQI was administered to the individuals with vestibulopathy in order to verify possible abnormalities in the quality of sleep. After analysis, it was evidenced that the majority (38 individuals, 73.07 %) of the 52 individuals with vestibulopathy demonstrated poor quality of sleep as shown by their global PSQI scores. Significant differences were obtained in six out of seven components investigated by the questionnaire, namely quality, latency, duration, efficiency and disturbances of sleep, daytime dysfunctions in addition to the global score indicating the presence of sleep disorders in the majority of the individuals with vestibulopathy in the present study and separating the sample into two groups. Until the present moment, no other studies were found in the relevant literature in which the PSQI was administered or reporting sleep disorders in individuals with vestibulopathy, not only qualifying the present study as original but also with limited possibilities for comparison of epidemiological findings.

The Dizziness Handicap Inventory (DHI) was administered in order to evaluate the interference of dizziness in the perceived quality of life in individuals with vestibulopathy. This questionnaire investigates the impact of the dizziness when executing certain eye, head and body movements while performing professional, domestic, social and leisure activities and in the level of independence to perform these movements, such as unassisted ambulation.^19^ It was observed in the present study a deficit in the quality of life of individuals with vestibulopathy in both groups, similar to previous reports.^29^, ^30^, ^31^ The majority of patients with vestibular dysfunctions ad dizziness restrict their physical activities and traveling in an attempt to decrease the risk of unpleasant symptoms.^29^ In addition, these individuals show less independence in their personal care, instrumental and social activities outside the house, work and family life.^32^

The group with vestibulopathy and poor quality of sleep demonstrated worse perceived quality of life in the physical aspect and in total DHI score with significant results when compared to the group with good quality of sleep. In the emotional and functional aspects the group with poor quality of sleep also showed worse perceived quality of life albeit without statistical significance. No other studies were found in the literature that reported administering the DHI to patients with vestibulopathies and sleep quality disorders, which makes it difficult to compare the present findings with similar samples.

The Fall Index generated by Tetrax can be used to predict de ability of ambulate and measure independence.^33^ In the present study high Falls Indexes were observed in the group with vestibulopathy and poor quality of sleep while the group with vestibulopathy and good quality of sleep showed moderate risk of falls and the control group, low risk. Only between the group of individuals with vestibulopathy and poor quality of sleep and the group of healthy individuals statistically significant differences were noted in this study. Therefore, it can be hypothesized that the group of individuals with vestibulopathy and good quality of sleep may be considered intermediate or borderline between the healthy controls and the individuals with vestibulopathy and poor quality of sleep with regards to the Fall Index.

In the final model of this study the variables quality of sleep and quality of life remained as predictive factors of occurrence of moderate/high risk of falls, probably because poor quality of sleep may have an effect on the vestibular system, decreasing its function and eventually the perception of the body movement in space, in addition to asthenia and physical fatigue which could increase the risk of falls.^5^, ^34^ In the same manner, worse quality of life may provoke decrease in the ability to concentrate, to focus attention and to remember facts, also contributing to a higher risk of falls.^35^ Poor quality of sleep causes loss of concentration and memory, irritability, decreased ability to perform tasks of daily life, decreased pleasure in the social and family relationships as well as sleepiness, lack of motivation and depressive mood,^34^ which may explain the variable quality of sleep as a predictive factor of worse quality of life in individuals with vestibulopathy.

The posturography assessment performed with the Tetrax Interactive Balance System (Tetrax®) evidenced abnormalities in the evaluated parameters of postural control in the individuals with vestibulopathy with good and poor quality of sleep. In this study significant differences were obtained in the Fall Index and global stability score in individuals with vestibulopathy when the group with poor quality of sleep was compared with the control group. The group with vestibulopathy and good quality of sleep seemed to perform as an intermediate group. This may have happened due to the fact this group presents less sleep disturbances, therefore suffering less with the harmful effects of them such as muscle fatigue, loss of concentration and increased deficit in the vestibular function,^5^, ^36^ which probably enabled better postural control. The data obtained regarding the length of diagnosis showed the chronicity of the disease in this sample and lack of vestibular compensation, since all the individuals with vestibulopathy reported dizziness. The group with vestibulopathy and poor quality of sleep showed worse performance not only in the perceived quality of life but also in all of the posturography scores. This leads to the belief that the quality of sleep may influence the vestibular system function and other mechanisms responsible for maintaining balance, causing abnormalities in the postural control and, consequently, in the quality of life of individuals with vestibulopathy. These interferences were evidenced by the positive correlation amongst data and also by the linear and logistic regression applied in the present study.

Lastly, the identification of peculiarities in the balance disorders and their interaction with other disorders in individuals with peripheral vestiulopathies may have positive diagnostic, preventative and therapeutical implications, since falls are the most common cause of death in elderly^17^; studies such as this are important in determining aspects to be improved to prevent falls in this group.

The investigation of the quality of sleep in all patients with chronic non- compensated vestibulopathy become important after the present findings, leading to the possibility of further research. New models of research with more objective methods of sleep assessment should be designed to better clarify its influence in the balance disorder, in the parameters of the postural control assessed by the posturography and all the intercorrelations that may affect negatively individuals with peripheral vestibulopathies.

## Conclusion

Individuals with peripheral vestibulopathies and poor quality of sleep demonstrate worse balance evidenced by increased postural instability, higher risk of falls and worse perceived quality of life.

The quality of sleep is a predictive factor for worse perceived quality of life and for higher risk of falls in individuals with peripheral vestibulopathies.

## Funding

This study was supported by CAPES.

## Conflicts of interest

The authors declare no conflicts of interest.
